# Supplementary material for: Pregnant women’s attitudes and intentions toward tuberculosis, malaria, group B streptococcus, and respiratory syncytial virus vaccines in pregnant: Findings from a cross-sectional study of pregnant women living in Brazil, Ghana, Kenya, and Pakistan
Source: PLOS Glob Public Health. 2026 Jan 28;6(1):e0004562. doi: 10.1371/journal.pgph.0004562 (PMC12851439; doi:10.1371/journal.pgph.0004562)
Supplement: Attachment — Submitted filename: Review.docx [file pgph.0004562.s001.docx]

In this study Rupali et al have explored the attitudes of pregnant women in 4 countries regarding the uptake of 4 plausible future vaccines. The topic is an important one and the study has been conducted well. Please find my comments and queries below.

Major comments

1. The study has been described as a mixed methods study. However, only quantitative surveys seem to have been conducted. Were there no interviews or focus group discussions done to understand the major emerging themes regarding the attitudes of the participants?
2. Why was knowledge on COVID-19 vaccine a requirement for inclusion in the study?
3. Of the many potential vaccine- preventable diseases, why these specific four vaccines were selected for the purpose of the study?
4. Why was the data collected using Likert scale dichotomized during analysis? This could have led to potential loss of information.
5. It is not clear from the methods section whether any information on the potential effects of these 4 diseases on pregnancy was imparted to the participants. The attitude of the study participants will definitely depend upon their perception of threat from each of these diseases for themselves as well as the baby. It is difficult to imagine that all the study participants had baseline knowledge about diseases caused by GBS or RSV. Even in the case of more well-known diseases like malaria or TB, their effect on pregnancy is not something which is widely known. Please explain.
6. In Discussion section (lines 404 to 408) the authors are attributing the favorable attitude of pregnant women for GBS vaccine to the high prevalence of this infection, antibiotic resistance and the disease related morbidity and mortality. Again, unless the information regarding these was provided to the participants, it is difficult to assume that these factors would have influenced their choices.

Minor comments

1. Please correct the repetition in the following statement found in abstract *“Participants indicated that vaccine safety for the baby was the most important factor in their decision-making related to vaccine acceptance, followed by vaccine efficacy for the baby, and then vaccine safety for the baby*.”
2. Please rephrase and correct the sentence “The sample size was determined with 80% power to find differences in proportions of respondents with an attitude between two groups with a 5% margin of error.” Proportion with **an** attitude??
3. Line number 200 is confusing. “members of the team from (blinded for review) through…”
4. “In Kenya, vaccine safety for the mother was chosen most frequently, and in Ghana, vaccine efficacy for the mother was chosen most frequently.” Use of the conjunction AND seems inappropriate here.
5. In lines 360 to 362 the use of terms like “least likely to definitely intend to receive” is very confusing to read.
