# Supplementary material for: Pregnant women’s attitudes and intentions toward tuberculosis, malaria, group B streptococcus, and respiratory syncytial virus vaccines in pregnant: Findings from a cross-sectional study of pregnant women living in Brazil, Ghana, Kenya, and Pakistan
Source: PLOS Glob Public Health. 2026 Jan 28;6(1):e0004562. doi: 10.1371/journal.pgph.0004562 (PMC12851439; doi:10.1371/journal.pgph.0004562)
Supplement: Attachment — Submitted filename: Response to Reviewers FVI.docx [file pgph.0004562.s003.docx]

Reviewer #1: Thank you for asking me to review this paper which reports the findings of a cross-sectional survey, completed by pregnant women in four countries on their attitudes towards vaccinations. The paper reports an important area of study and I applaud the authors for their important message about the importance of inclusion of pregnant women in clinical trials. However, there are several issues I have identified in the paper, mostly around methods and expansion on why some of the questions have been asked, which would require revision prior to publication.

Title
• Title should include that this is a cross-sectional survey, i.e. “Findings from a cross-sectional survey of pregnant women…..”

Thank you for this suggestion, we have modified the title.

Background
• Worth mentioning in the background that GBS can also result in death of the baby

Thank you for this suggestion, we have edited the background accordingly.

• Background sets the scene nicely but would be helpful to expand a little on the final paragraph re women’s attitudes to vaccines – briefly mention any previous work and why this is an important question to answer, i.e. even if vaccines are made available in LMICs important to know whether they would be considered acceptable to those who they are intended for

Thank you for this suggestion, we have edited this paragraph accordingly.

• As this paper reports the results of a cross-sectional survey, as part of a wider programme of work, this should be specified at the end of the background section, rather than in the ‘participants, study setting and recruitment’ sub-section of Methods. It will make it clear that this study reports only the survey data, and that data collected from qualitative interviews is reported separately.

Thank you for this suggestion, we agree and have added in this clarification to the end of the introduction section as suggested.

• The background section should include a clearly framed research question, with clearly specified objectives for the study.

Thank you for your comment. We have sought to clarify our study objectives.

Methods
• Please add a sentence that summarises the total number of sites/facilities that were involved

We have added a summary sentence of the number of facilities included in the study.

• Page 7, the sentence regarding sample size is unclear – ‘proportion of respondents with an attitude’. Surely everyone had an attitude, positive or negative, towards vaccines. Please be clearer here. What were the sample size assumptions based on?

Thank you for this suggestion. We have added language to clarify this as the questions that the sample size was based on are binary, with either a positive or negative view of vaccines in pregnancy.

• It’s unclear to me why formal statistical testing was undertaken and looking for a statistical difference between countries. For this kind of study, I would have expected to see descriptive statistics only reported.

This study did not perform any formal hypothesis testing as part of this descriptive analysis. Table 2 contains the only results that report specific p-values, and the purpose of these is to compare attitudes across countries. Demand generation activities are not universal and are best tailored to the target population or region. We have added a statement to the objectives of the study at the end of the introduction to stress this point and justify the comparison between countries.

• Data collection – were women provided with written participant information? Please make this clear

All participants were provided with written participant information sheets during the consenting process. This has now been added to the methods.

• Please explain the rationale for having “heard of Covid-19 vaccine” as an eligibility criteria for inclusion

The analysis presented in this manuscript is part of a larger study focused on surveying pregnant women about COVID-19 vaccine attitudes, beliefs, intentions, and behaviors. We included these questions about future maternal vaccines within a larger survey about COVID-19 vaccination in pregnancy, which is why that is included as inclusion criteria. We have added some clarity about this in the methods section.

• The countries who provided food box/transport renumeration – was this before or after completion of the survey?

All remuneration was provided after the survey was administered. This has been added to the methods.

• Future maternal vaccine intentions: Did you collect data on the reasons for the response choice for the question relating to their intentions? i.e. “I would not have no intentions of receiving the vaccine”…. Because……-

Please see the subsection “Decision-making factors related to future maternal vaccines” in the Methods section. Participants were asked to rank the most and least important factors informing their decision to accept a new maternal vaccine. In subsection, “Priority maternal vaccine and decision-making" of the Methods, we asked participants to rank reasons why they chose the future vaccine they are most interested in receiving. These results are presented in Figure 1 and Figure 4. While we did not ask the specific reason that participants had no intention to receive the vaccine, these other questions give some insight on the specific characteristics of a vaccine (safety, efficacy, doctor and family recommendation) that women prioritize.

• Vaccine hesitancy – please explain the rationale for including a very specific question relating to the Covid-19 vaccination. I appreciate this is likely to set the scene since many women will have heard of this particular vaccine, but I think some context is required. Arguably, getting a vaccine for Covid-19 is quite different to GBS, for example.

Thank you for this comment. We focused on COVID-19 as this was part of a larger study that focused on the decision-making process related to COVID-19 vaccination for pregnant women. We have included this information in the methods and have noted it as a limitation.

Reviewer #2: Line 44 to 46: vaccine safety for baby has been repeated,

Thank you for this comment. This was a typo as safety for the mother was the third most important factor. We have edited this.

Line 157: you have mentioned COVID 19 vaccine decision making

Thank you for this comment. This line’s mention of COVID-19 vaccines is intentional, as the sentence describes how this manuscript’s analysis is part of a larger study that was originally focused on pregnant women’s COVID-19 vaccine decision-making. The COVID-19 vaccine decision-making survey we administered included a section on other future maternal vaccines, and the analysis of that section is what constitutes this paper. We have revised the methods to further clarify this.

Line 188: why having heard of the COVID-19 vaccine is an inclusion criteria? Isn't the study about TB, Malaria, GBS and RSV?

As discussed above, the analysis presented in this manuscript is part of a larger study focused on surveying pregnant women about COVID-19 vaccine attitudes, beliefs, intentions, and behaviors. We included these questions about future maternal vaccines within a larger survey about COVID-19 vaccination in pregnancy, which is why that is included as inclusion criteria. We have added some clarity about this in the methods section.

Line 259: same as above, the vaccine hesitancy section is all about COVID-19

As stated, this analysis is from a larger survey on COVID-19 vaccine attitudes, beliefs, intentions, and behaviors among pregnant women. While the larger survey was focused on COVID-19 vaccines, we also asked the same sample of women about future maternal vaccines (TB, Malaria, GBS, RSV).

Line 373: While the study provides useful insights into vaccine prioritization among pregnant women across countries, it remains unclear whether participants had adequate knowledge of the diseases or vaccine safety profiles. The absence of questions exploring their rationale limits the interpretability of these preferences. Most women have received high school education, when presented with a list of 4 vaccines, it is overwhelming and their choices reflect guesswork or based on whatever little they know or don't know about that disease. Without adequate knowledge about the disease, it's burden and consequences being informed, it is difficult to understand the rationale to understand vaccine acceptance study 4 different diseases.

Thank you for this important observation. In our survey, which is available through Open Science Framework (the citation has been added to the manuscript), we briefly describe both RSV and GBS diseases, which are not nearly as well-known as TB and malaria. Because these vaccines are (mostly) still underdevelopment, we were not able to describe any safety profiles. However, when women were asked about their intention to receive each of the vaccines, they were asked under the premise that the vaccine was licensed for use in pregnancy, available, and that their doctor recommended it.

Reviewer #3: In this study Rupali et al have explored the attitudes of pregnant women in 4 countries regarding the uptake of 4 plausible future vaccines. The topic is an important one and the study has been conducted well. Please find my comments and queries below.

Major comments

1. The study has been described as a mixed methods study. However, only quantitative surveys seem to have been conducted. Were there no interviews or focus group discussions done to understand the major emerging themes regarding the attitudes of the participants?

The questions about future vaccines for use in pregnancy were included in the quantitative survey portion of the study only, hence the inclusion of the survey data only. We have clarified this in the methods.

2. Why was knowledge on COVID-19 vaccine a requirement for inclusion in the study?

The analysis presented in this manuscript is part of a larger study focused on surveying pregnant women about COVID-19 vaccine attitudes, beliefs, intentions, and behaviors. We included these questions about future maternal vaccines within a larger survey about COVID-19 vaccination in pregnancy, which is why that is included as inclusion criteria. We have added some clarity about this in the methods section.

3. Of the many potential vaccine- preventable diseases, why these specific four vaccines were selected for the purpose of the study?

Thank you for this question. We have added language to clarify the selection of these four vaccines for this analysis. The four vaccines selected for this analysis — within the overarching study of maternal COVID-19 vaccination — were chosen because they have been identified as high priority vaccines for use in pregnancy but have not yet been licensed or introduced in low- and middle-income settings. Maternal RSV vaccines are licensed and recommended by WHO for introduction, but to date are primarily available in higher income settings. Maternal GBS vaccines are under development (phase 2 and 3 trials) and are expected in the next 5-10 years. These two vaccines are developed specifically for use in pregnancy given the nature of the diseases they target. Currently available TB and malaria vaccines are not recommended for use in pregnancy but new malaria vaccines have recently been studied in pregnancy and TB vaccine candidates are a priority for studying use in pregnancy. While other vaccines are of interest in pregnancy, these four have been identified as priorities for LMIC settings given the high burden of disease and particular severity and incidence in pregnancy and among neonates.

4. Why was the data collected using Likert scale dichotomized during analysis? This could have led to potential loss of information.

The distribution of responses to all 4-point Likert scale questions were first explored before dichotomizing. Across all of these questions included in this analysis (vaccine hesitancy and family influence), the most extreme responses (strongly agree or strongly disagree) had the smallest counts with the vast majority of answers in the agree and disagree groups. Given the descriptive nature of this study, we felt confident that dichotomizing these responses would not change our conclusions while also making the results more readibly interpretable.

5. It is not clear from the methods section whether any information on the potential effects of these 4 diseases on pregnancy was imparted to the participants. The attitude of the study participants will definitely depend upon their perception of threat from each of these diseases for themselves as well as the baby. It is difficult to imagine that all the study participants had baseline knowledge about diseases caused by GBS or RSV. Even in the case of more well-known diseases like malaria or TB, their effect on pregnancy is not something which is widely known. Please explain.

Thank you for this comment. Our intention with this component of the survey was to conduct an exploratory analysis describing how participants — pregnant and lactating women in the four study countries — perceive these diseases and potential vaccines in pregnancy at baseline, without extensive information provided about specific disease impacts in pregnancy or potential vaccines. We sought to understand the current preferences among participating women and to explore how these may be associated with other KAB factors explored in this broader survey focused on COVID-19 vaccination in pregnancy. We agree that pregnant women’s decision to accept or reject a vaccine in pregnancy hinges on a number of factors that are both cross-cutting (disease agnostic) and disease-specific, as well as related to their own health and that of their babies, and that the risk and benefit assessment for a specific vaccine will vary. For this descriptive study, we aimed to document these perspectives as they relate to future maternal vaccines and identify areas for further research.

Before beginning this battery of questions in the survey, data collectors read the following script: *“In this last section, I will ask you about your feelings on vaccinations during pregnancy in general and about new vaccines that may become available for pregnant people in the future. For example, there are currently vaccines in development for tuberculosis, malaria, group B streptococcus (bacteria that can cause blood infections or meningitis in babies), and respiratory syncytial virus or RSV (a virus that causes wheezing and pneumonia in young children). There are no right or wrong answers.”* Each question asked participants to This information, along with the full survey instrument, is available in the published protocol manuscript and file repository (https://osf.io/2kmdw ) and cited in our methods section accordingly.

We have added language to the limitations section to acknowledge this.

6. In Discussion section (lines 404 to 408) the authors are attributing the favorable attitude of pregnant women for GBS vaccine to the high prevalence of this infection, antibiotic resistance and the disease related morbidity and mortality. Again, unless the information regarding these was provided to the participants, it is difficult to assume that these factors would have influenced their choices.

Thank you for this comment, we have refined our discussion language to more accurately reflect our interpretation of these GBS findings and to highlight opportunities for further exploration.

Minor comments
1. Please correct the repetition in the following statement found in abstract “Participants indicated that vaccine safety for the baby was the most important factor in their decision-making related to vaccine acceptance, followed by vaccine efficacy for the baby, and then vaccine safety for the baby.”

Thank you for flagging this. We have fixed the sentence so that it now reads “Participants indicated that vaccine safety for the baby was the most important factor in their decision-making related to vaccine acceptance, followed by vaccine efficacy for the baby, and then vaccine safety for the mother.”

2. Please rephrase and correct the sentence “The sample size was determined with 80% power to find differences in proportions of respondents with an attitude between two groups with a 5% margin of error.” Proportion with an attitude??

Thank you for this suggestion, this section has been amended to clarify that the difference in attitude means the difference in the proportion of the respondents with a positive vs negative view towards vaccines in pregnancy.

3. Line number 200 is confusing. “members of the team from (blinded for review) through…”

Thank you for noting this error. We had initially submitted the manuscript to the journal in a double-blinded format, which is why the name of the institute was redacted with “(blinded for review).” As the review process is not double-blinded, we have replaced the redaction with the correct institution.

4. “In Kenya, vaccine safety for the mother was chosen most frequently, and in Ghana, vaccine efficacy for the mother was chosen most frequently.” Use of the conjunction AND seems inappropriate here.

Thank you for this suggestion. We have replaced the “and” with “while.”

5. In lines 360 to 362 the use of terms like “least likely to definitely intend to receive” is very confusing to read.

We have edited these sentences to reduce confusion.
